# Mechanisms of Pathogenesis, Infective Dose and Virulence in Human Parasites

**DOI:** 10.1371/journal.ppat.1002512

**Published:** 2012-02-16

**Authors:** Helen C. Leggett, Charlie K. Cornwallis, Stuart A. West

**Affiliations:** Department of Zoology, Oxford University, Oxford, United Kingdom; Emory University, United States of America

## Abstract

The number of pathogens that are required to infect a host, termed infective dose, varies dramatically across pathogen species. It has recently been predicted that infective dose will depend upon the mode of action of the molecules that pathogens use to facilitate their infection. Specifically, pathogens which use locally acting molecules will require a lower infective dose than pathogens that use distantly acting molecules. Furthermore, it has also been predicted that pathogens with distantly acting immune modulators may be more virulent because they have a large number of cells in the inoculums, which will cause more harm to host cells. We formally test these predictions for the first time using data on 43 different human pathogens from a range of taxonomic groups with diverse life-histories. We found that pathogens using local action do have lower infective doses, but are not less virulent than those using distant action. Instead, we found that virulence was negatively correlated with infective dose, and higher in pathogens infecting wounded skin, compared with those ingested or inhaled. More generally, our results show that broad-scale comparative analyses can explain variation in parasite traits such as infective dose and virulence, whilst highlighting the importance of mechanistic details.

## Author Summary

We found that mechanisms used by parasites to infect hosts are able to explain variation in two key pathogen traits: infective dose and virulence. In pathogens where the molecules secreted to facilitate infection acted locally, the number of cells required to start an infection (infective dose), was lower than in pathogens where the secreted molecules act more distantly. Parasite virulence showed no correlation with local versus distant action, but was negatively correlated with infective dose, and greater in species that infect via wounded skin. By showing how such parasite life history details matter, our results help explain why classical trade-off models have been relatively unsuccessful in explaining broad scale variation across parasite species.

## Introduction

There is huge variation across pathogen species in the number of cells required to successfully infect a host. This number is known as the ‘infective dose’. At one end of the scale, species such as *Shigella* and *Giardia lamblia* require about 10 cells to start an infection. In contrast, species such as *Vibrio cholera* and *Staphylococcus aureus* require 10^3^–10^8^ cells in order for an infection to develop [1]–[3]. It is unclear why infective dose varies, with large differences occurring even between closely related pathogens [2], [3].

Schmid-Hempel and Frank [2] predicted that the variation in infective dose could be explained by the different biochemical mechanisms that pathogens use to infect hosts. Pathogens secrete a number of molecules which facilitate the suppression and/or evasion of host immune responses, and hence aid parasite growth. If these molecules act locally, in the vicinity of the pathogenic cell, then only small numbers of molecules may be required for successful growth and so infections can be established from small numbers of pathogenic cells. In contrast, if the pathogenic molecules diffuse and therefore act at a distance, then large numbers of molecules may be required for evading the host immune system. In these cases greater numbers of pathogenic cells could be needed to establish an infection. However, while this prediction is consistent with anecdotal data [2], [3], it has yet to be tested formally.

Here, we test Schmid-Hempel and Frank's [2] prediction that infective dose is determined by whether pathogenesis is locally or distantly acting. We use data from 43 species of human pathogens across a range of enteropathogenic bacteria, protozoa, fungi and viruses. A possible problem with comparative studies across species is that closely related species can share characters through common descent rather than independent evolution. Consequently, analysing species as independent data points can lead to misleading correlations [4]–[6]. For example, all viruses are locally acting, and so this could lead to patterns between viruses and bacteria, rather than local or distant action. We account for this potential problem of shared ancestry by using multivariate nested taxonomic models [7], [8].

We then extend this work in two ways. First, Schmid-Hempel and Frank [2], [3], [9] further predicted that pathogens with distantly acting immune modulators will be more virulent, possibly because they would have a large numbers of cells in the inoculums, and higher parasite density would overwhelm the host immune system causing more harm to hosts. We therefore test whether the virulence of pathogens with distantly acting immune modulators is greater than that of pathogens with locally acting molecules. Second, we test the influence of two other factors that could affect infective dose and virulence: mode of transmission (direct or indirect) and route of infection (ingestion, inhalation or wounded skin) [1], [10]–[12]. These factors could influence dose and virulence for a number of reasons, including their affect on: the extent to which virulence reduces pathogen transmission; the types of immune response they encounter; and the genetic diversity (or relatedness) of the pathogens either competing for or cooperating to exploit the host [2], [3], [9]–[28].

## Results/Discussion

**Figure 1:**
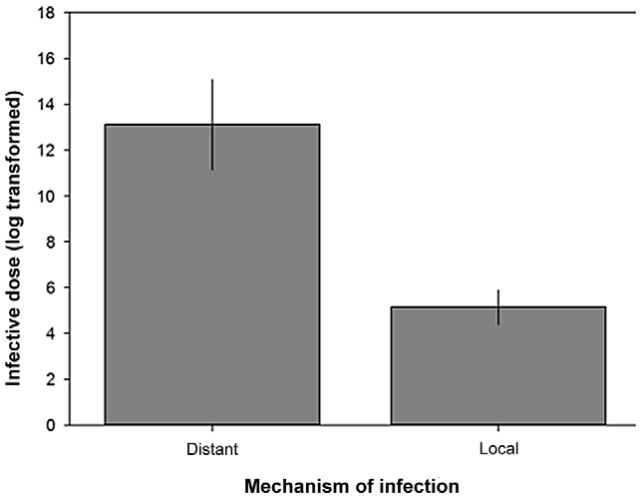
Infective dose and the mechanisms used by pathogens to infect hosts. Means ±1 standard error (Table S2 in Text S1).

We found that pathogens with immune modulators that act distantly within the host have significantly higher infective doses than pathogens with locally acting molecules, (Figure 1 and Table S2 in Text S1: F_1, 40_ = 25.79, P<0.0001). This supports the prediction by Schmid-Hempel and Frank [2] that local pathogenic action requires only a small number of molecules, and thus relatively few cells are needed to start an infection, compared to distantly acting mechanisms where a large number of diffusible molecules need to accumulate in order to overwhelm the host's immune clearance.

**Figure 2:**
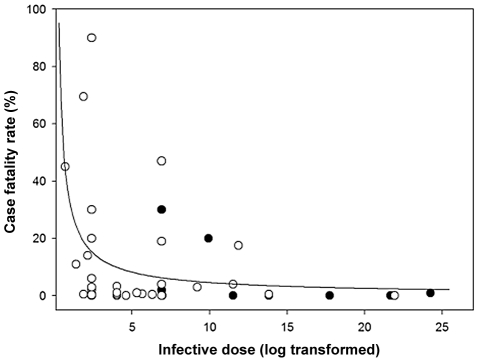
Variation in case fatality rate explained by infective dose. Means ±1 standard error. Line represents a logistic curve (Table S3in Text S1).

Contrary to the hypothesis that pathogens with distantly acting immune modulators are more virulent, we found no significant relationship between case fatality rate or severity of infection and the mechanism of pathogenesis (Table S3 and Table S4 in Text S1: P>0.05). However, case fatality rate was significantly negatively related to infective dose of pathogens (Figure 2 and Table S3 in Text S1: F_1, 38_ = 3.94, P = 0.05). We suggest this correlation arises because for a given dose, pathogens that are locally acting and have lower infective doses are more likely to establish an infection. For this relationship to hold, we reasonably assume that the actual dose in natural infections is largely determined by factors such as mode of transmission, and so does not show a strong covariance with whether a parasite acts locally or globally within the host. We attempted to collect data on mean parasite dose in different transmission modes during natural infections so we could examine how this correlates with local/global within-host parasite action, but we were unable to obtain sufficient data.

There are at least two possible alternative explanations for the negative relationship between case fatality rate and infective dose of parasites, although we suggest these are less likely than the above explanation. First, recent theory suggests parasites might adapt to low infective doses by evolving a higher per-parasite growth rate, causing greater host exploitation and virulence [29]. However, the reduction in dose in this model results from increased host resistance, and there is no reason to assume that selection for host resistance consistently differs between global and local acting parasites.

Second, a low infective dose may reduce the incidence of multiple genotype pathogen infections since there are fewer parasites in the inoculum, which could favour higher levels of cooperation between parasites, and hence lead to greater growth and virulence [24], [25]. However, the extent to which this will be of general importance will be limited by the fact that different biological details can lead to different relationships between strain diversity and virulence. For example, when different parasite strains compete for host resources, higher strain diversity is expected to lead to greater virulence [10], [11], [22], [29]–[33]. Alternatively, antagonistic interaction between strains, such as chemical warfare, can lead to a predicted domed relationship between strain diversity and virulence [26]. Nonetheless, it is possible that all three explanations could play a role, with their importance varying across species.

**Figure 3:**
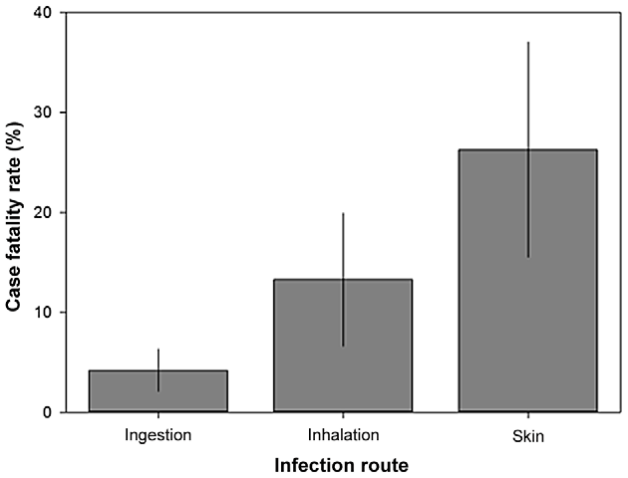
Variation in case fatality rate explained by infection route. Means ±1 standard error. Line represents a logistic curve (Table S3 in Text S1).

We found that pathogens infecting hosts through wounded skin result in significantly higher case fatality rates than pathogens inhaled or ingested (Figure 3 and Table S3 in Text S1: F_2, 26_ = 5.30, P = 0.01). Given that infection via wounded skin includes transmission via bites of insect vectors and contaminated water, this result supports theory on virulence-transmission trade-offs which proposes that vectors and water systems circumvent the need for an ambulatory host to transmit pathogens, selecting for the evolution of higher virulence [12], [18], [21]. However, another potentially important factor is that the type of immune response that pathogens are confronted with will affect virulence. Pathogens that infect hosts through wounded skin evade mechanical immunity and directly enter the circulatory system. Hence, they may cause virulent systemic infections more readily than ingested or inhaled pathogens, which must overcome other anti-infection barriers such as stomach acid and mucus membranes before causing systemic infections.

More generally, our results emphasise the importance of life-history or mechanistic details for the evolution of parasite traits. Theoretical models for the evolution of parasite traits such as virulence have generally relied on simple trade-offs between virulence and transmission. These models have been able to explain variation in virulence both within species, and between closely related species with similar life histories [15], [20], [28], [33]–[37]. In contrast, this body of theory has been less successful at explaining broad scale variation across species [2], [9], [25], [27]. One possible explanation for this is that the predictions of virulence theory can depend upon the mechanisms that parasites use to infect and exploit hosts, which are not considered in the classical models; hence our expectations of data fitting the model may be too high. If the details of how parasites infect hosts really matter, this would limit the extent to which we would expect to find broad empirical patterns to match theory [25]. Our results show that transmission, dose and virulence can be influenced by mechanistic details such as distance at which molecules act and route of infection.

## Materials and Methods

### Infective dose

We obtained data on the number of pathogen cells required to start an infection (infective dose) by searching: (a) databases from the United States Food and Drug Administration [38], Health Canada [39], Medscape [40], the Centre for Disease Control and Prevention [41], the World Health Organisation [42]; (b) empirical studies found via keyword searches in the ISI Web of Knowledge database [43]. Where ranges or more than one estimate of infective dose were given, we calculated the median infective dose to use in our analyses.

We emphasise that uncertainties exist in infective dose measurements: often they were extrapolated from epidemiologic investigations, were obtained by human feeding studies on healthy, young adult volunteers, or are best or worst estimates based on a limited data from outbreaks. Where known, we give methods of estimation in Table S1 in Text S1.

### Classifying mechanisms of pathogenesis

We classified pathogens as having local or distant action according to the framework of Schmid-Hempel and Frank [2]. For local action, pathogens directly interact with host cells via surface-bound molecules or by injecting proteins into host cells by a type III or IV secretion systems. For example, *Yersinia entricolata/tuberculosis* injects Yop protein into target cells via a type III secretion system, leading to cytotoxicity [44], and *Ebola virus* binds to different cell surfaces and replicates leading to cell necrosis [45].

For distant action, pathogens indirectly interact with host cells by secreting proteins that diffuse into their surroundings and only exert pathogenic effects when they bind to host cells. This may arise, for example, through immune modulators delivered by the general secretary pathway, or the type I, II and V secretary systems. For example, the well known virulence factor lysteriolysin O of *Listeria monocytogenes* and exotoxins of *Staphylococcus aureus*, are secreted via the general secretory pathway [46]. We do not classify between interactions with host and immune cells specifically since we are concerned with how far the interaction occurs from the infecting parasite, not with what cell the interaction occurs with.

### Measuring virulence: Case fatality rate, ‘disease severity’ and incidence

To capture both the short and long term consequences of pathogen infection on host health, we use case fatality rate and a ‘disease severity’ score to measure pathogen virulence. These are two of the three criteria used to estimate ‘burden of disease’ in a recent protocol for prioritising infectious disease in public health [47].

We rated each pathogen according to its severity, as described in Table 1. We gave a score of 0 to pathogens of average importance, or pathogens for which a lack of data precluded another score. Incidence data are the estimated mean number of new cases per year in the USA. Case fatality rates are estimates of fatality without treatment or co-morbidities and represent the number of cases of a disease ending in death compared to the number of cases of the disease. We obtained data from the before-mentioned databases, plus various reports in the literature (see Table S1 in Text S1).

**Table 1:** Definition of the scores for calculating disease severity.

Scores		
−1	0	1
Hospitalisation is rare. Work loss is <2 days. No persisting illnesses/handicaps	Hospitalisation is rare. Work loss >5days is rare. Few persisting illnesses/handicaps.	Hospitalisation is frequent, work loss of >5days is frequent. Persisting illnesses/handicaps occur.

Adapted from [47].

We emphasise that while a “case” should represent an infected individual, in practice it may involve infection of some severity, -hospitalization even. Thus overall our definition of case fatality may overestimate virulence. For example, a benign parasite that infects many hosts asymptomatically, but cause severe disease in a small proportion of hosts, may be classified as virulent. By contrast, a virulent parasite that causes disease of equal severity in its hosts may be classed as less virulent. To correct for this potential bias, we assessed whether case fatality rate is linked to incidence rate, and examined the effects of the other variables after controlling for variation in incidence rate.

### Transmission mode and route of infection

We obtained data on transmission mode and route of infection using the before-mentioned databases. We classified pathogens as either direct or indirectly transmitted: direct transmission requires physical contact between an infected and susceptible host, and indirect transmission requires an agent to transfer the pathogen from an infected to a susceptible host. We classified the routes of infection used by pathogens as entry through wounded skin, inhalation, or ingestion. For example, *Bordatella pertussis* is usually spread by infected people coughing or sneezing while in close contact with susceptible others who then inhale the *pertussis* bacteria [41] (i.e. direct transmission). Where pathogens can use more than one mechanism of transmission or infection, we used the mechanism stated in the infective dose data for our analyses.

### Statistical analysis

We performed three analyses. First, we tested whether minimum infective dose (log transformed) was related to the mechanism of infection (2 level fixed factor: local or distant), infection route (3 level fixed factor: ingestion, inhalation, wounded skin) and the transmission mode of pathogens (2 level fixed factor: direct, indirect) using a linear mixed effects model (LMM) with restricted maximum likelihood estimation (REML). Second we tested if case fatality rate (% of cases resulting in death) was influenced by infective dose (covariate log transformed), incidence (covariate log transformed), mechanism of infection, infection route and transmission mode using a generalised linear mixed effects model (GLMM) with a binomial error distribution. Finally, we analysed the severity of infection (−1, 0, 1) in relation to the same explanatory variables as the second analysis using a GLMM with an ordered multinomial error distribution.

The data (Table S1 in Text S1) encompass a diverse range of pathogens. We obtained information on the taxonomic classification of pathogens from the National Center for Biotechnology Information (NCBI) [48]. We accounted for the non-independence of data arising from phylogenetic relationships between pathogens in all LMMs and GLMMs using nested taxonomic random effects structures whereby each taxonomic level (genus, order, class and kingdom) was nested within all higher taxonomic levels (see Tables S2–4 in Text S1 for details). We only entered genus, order, class and kingdom into models because of poor replication at other taxonomic levels. We examined the significance of fixed effects (factors and covariates) using Wald type adjusted *F* statistics and the effect with the highest P value was sequentially dropped until only significant terms (P<0.05) remained [49]. Prior to all analyses covariates were Z-transformed (mean = 0, standard deviation = 1). We used the Kenward and Roger (1997) method for estimating standard errors for parameter estimates and denominator degrees of freedom since it is specifically designed for models with multiple random effects and unbalanced data, increasing the accuracy of significance tests [50]–[52]. We assessed the significance of random effects using log-likelihood ratio tests (LRTs) [53]. All analyses were conducted in SAS version 9.2.

## Supporting Information

Text S1 **Dataset and statistical analysis tables.** Here we provide details of the pathogens included in this study and summaries of the statistical analysis: Table S1: Pathogens included in the analysis; Table S2: LMM of infection dose; Table S3: GLMM of case fatality rate; Table S4: GLMM of severity of infection.
(DOC)
