# Supplementary material for: Mechanisms of Pathogenesis, Infective Dose and Virulence in Human Parasites
Source: PLoS Pathog. 2012 Feb 16;8(2):e1002512. doi: 10.1371/journal.ppat.1002512 (PMC3280976; doi:10.1371/journal.ppat.1002512)
Supplement: Text S1 — Dataset and statistical analysis tables. Here we provide details of the pathogens included in this study and summaries of the statistical analysis: Table S1: Pathogens included in the analysis; Table S2: LMM of infection dose; Table S3: GLMM of case fatality rate; Table S4: GLMM of severity of infection. (DOC) [file ppat.1002512.s001.doc]

**Supporting Information**

**Contents**

Table S1: Pathogens included in the analysis

Table S2: LMM of infection dose

Table S3: GLMM of case fatality rate

Table S4: GLMM of severity of infection

| **Table S1:** Mechanisms of pathogenesis, infective dose and virulence of human pathogens | | | | | | | | | | | | | | |  | | | |
| --- | --- | --- | --- | --- | --- | --- | --- | --- | --- | --- | --- | --- | --- | --- | --- | --- | --- | --- |
| **Pathogen** | **Mechanism of pathogenesis** | | **Infective dose (number of infective particles)** | **Severity** | | **Case Fatality Rate (%)** | **Incidence**  **(number cases/year USA)** | | **Route of infection** | | **Transmission mode** | | | | **Sources** | | |  |
| **Viruses** | |  |  | |  |  |  | |  | |  | | | |  | | |  |
| *Ebola virus* | | Local | 5.5 | | 1 | 69.5 | 0 | | Inhalation | | Direct | | | | [1-4] | | |  |
| *Norovirus* | | Local | 55 | | 0 | 0.075 | 14,250,000 | | Ingestion | | Direct | | | | [1,5,6] | | |  |
| *Polio viruses* | | Local | 3* | | 1 | 11 | 0 | | Ingestion | | Direct | | | | [1,7,8] | | |  |
| *Rotavirus Group A* | | Local | 55 | | 0 | 0.65 | 2,850,000 | | Ingestion | | Indirect | | | | [1,9-11] | | |  |
| *Variola minor* | | Local | 55 | | 0 | 0.5 | 0 | | Inhalation | | Direct | | | | [8,12,13] | | |  |
| **Bacteria** | |  |  | |  |  |  | |  | |  | | | |  | | |  |
| *Bacillus anthracis* | | Distant | 20,250 | | 1 | 20 | 1 | | Skin | | Direct | | | | [1,4,9,14-16] | | |  |
| *Bacillus cereus* | | Distant | 1,000,000 | | -1 | 0 | 27,360 | | Ingestion | | Indirect | | | | [4,9,17-20] | | |  |
| *Bordetella pertussis* | | Local | 200 | | 1 | 1 | 6,000 | | Inhalation | | Direct | | | | [21-23] | | |  |
| *Brucella abortus* | | Local | 55 | | 1 | 1.025 | 150 | | Skin | | Direct | | | | [1,9,24] | | |  |
| *Brucella Melitensis* | | Local | 55 | | 1 | 3.25 | 150 | | Ingestion | | Indirect | | | | [1,9,25] | | |  |
| *Campylobacter jejuni* | | Local | 550* | | 1 | 0.4 | 2,850,000 | | Ingestion | | Indirect | | | | [1,4,8,9,15,20,26] | | |  |
| *Clostridium perfringens* | | Distant | 100,000 | | -1 | 0.07 | 607,239 | | Ingestion | | Indirect | | | | [4,9,20,27,28] | | |  |
| *Coxiella burnetii* | | Local | 10* | | 1 | 3 | 51 | | Inhalation | | Indirect | | | | [4,15,23,29] | | |  |
| *Cryptosporidium parvum* | | Local | 5.5 | | -1 | 0.5 | 1,529,000 | | Ingestion | | Indirect | | | | [1,4,9,26,30,31] | | |  |
| *Escherichia coli, enteroaggregative (EAEC)* | | Distant | 50,500,000* | | 0 | 0.025 | - | | Ingestion | | Indirect | | | | [26,32] | | |  |
| *Escherichia coli, enterohemorrhagic (EHEC, serotype O157)* | | Local | 10** | | 1 | 0.254 | - | | Ingestion | | Indirect | | | | [1,4,9,26,32] | | |  |
| *Escherichia coli, enteroinvasive (EIEC)* | | Local | 10* | | 0 | 0.025 | - | | Ingestion | | Indirect | | | | [4,9,26,32] | | |  |
| *Escherichia coli, enteropathogenic (EPEC)* | | Local | 3,367,000,000** | | 0 | 0.025 | - | | Ingestion | | Indirect | | | | [4,9,26,32] | | |  |
| *Escherichia coli, enterotoxigenic (ETEC)* | | Distant | 2,550,250,000* | | 0 | 0.025 | 48,710 | | Ingestion | | Indirect | | | | [1,4,9,26,32] | | |  |
| *Francisella tularensis, holarctica* | | Local | 1000 | | 1 | 0 | 200 | | Ingestion | | Indirect | | | | [9,33-35] | | |  |
| *Francisella tularensis, tularensis* | | Local | 7.5* | | 1 | 14 | 200 | | | Inhalation | | | | Indirect | | [1,4,9,15,33,34] | | |
| *Helicobacter pylori* | | Local | 10,000 | | 1 | 3 | 1,238,918 | | Ingestion | | | | Direct | | [4,23] | | |  |
| *Legionella pneumophila* | | Local | 140,000 | | 1 | 17.5 | 13,000 | | Inhalation | | | | Indirect | | [1,23,36-38] | | |  |
| *Listeria monocytogenes* | | Distant | 1000 | | 1 | 30 | 2,046 | | Ingestion | | | | Indirect | | [1,9,20] | | |  |
| *Mycobacterium tuberculosis* | | Local | 10 | | 0 | 0.021 | 14,517 | | Inhalation | | | | Direct | | [4,8,9,39] | | |  |
| *Neisseria gonorrhoeae* | | Distant | 1050 | | 1 | 0 | 700,000 | | Skin | | | | Direct | | [1,8,16] | | |  |
| *Pseudomonas aeruginosa* | | Local | 1000 | | 1 | 47 | 5,000,000 | | Skin | | | | Direct | | [40-42] | | |  |
| *Rickettsia conorii* | | Local | 10 | | 0 | 2.1 | 0 | | Skin | | | | Indirect | | [29] | | |  |
| *Rickettsia rickettsii* | | Local | 10 | | 1 | 20 | 1,125 | | Skin | | | | Indirect | | [1,4,29] | | |  |
| *Rickettsia prowazekii* | | Local | 10 | | 1 | 30 | 2 | | Skin | | | | Indirect | | [4,29,43] | | |  |
| *Salmonella enterica* | | Local | 283.75 | | 1 | 0.6 | 2,000,000 | | Ingestion | | | | Indirect | | [1,4,9,23] | | |  |
| *Salmonella paratyphi* | | Local | 1000 | | 1 | 4 | 659 | | Ingestion | | | | Indirect | | [4,8,9,20,44] | | |  |
| *Salmonella typhi (enterica typhimurium)* | | Local | 100,000 | | 1 | 4 | 1,100 | | Ingestion | | | | Indirect | | [1,4,8,44] | | |  |
| *Shigella flexneri* | | Local | 100 | | 1 | 0.1 | 206,549 | Ingestion | | | | Indirect | | | [1,4,8,9,13,15,20,26] | |  | |
| *Staphylococcus aureus* | | Distant | 100,000 | | 0 | 0.02 | 185,060 | | Ingestion | | | | Direct | | [9,20,30] | | |  |
| *Streptococcus A (pyogenes)* | | Local | 1000 | | 1 | 19 | 11,000 | | Inhalation | | | | Direct | | [1,9,45] | | |  |
| *Streptococcus pneumoniae (Group A)* | | Distant | 1000 | | 1 | 2 | 6,177,500 | | Inhalation | | | | Direct | | [1,8,9] | | |  |
| *Vibrio cholerae (serotypes O139, O1)* | | Distant | 33,334,000,000 | | 1 | 0.9 | 66 | | Ingestion | | | | Indirect | | [1,4,9,20,26] | | |  |
| *Yersinia enterocolitica* | | Local | 1,000,000 | | 0 | 0.5 | 67,243 | | Ingestion | | | | Indirect | | [1,4,20,46] | | |  |
| *Yersinia pestis* | | Local | 10 | | 1 | 90 | 13 | | Skin | | | | Indirect | | [1,29,47] | | |  |
| **Fungi** | |  |  | |  |  |  | |  | | | |  | |  | | |  |
| *Histoplasma capsulatum* | | Local | 10 | | 0 | 6 | 50,000,000 | | Inhalation | | | | Indirect | | [4,23,48,49] | | |  |
| **Protozoa** | |  |  | |  |  |  | |  | | | |  | |  | | |  |
| *Entamoeba histolytica* | | Local | 1 | | 1 | 45 | - | | Ingestion | | | | Direct | | [4,9,50,51] | | |  |
| *Giardia lamblia* | | Local | 10 | | 0 | 0.6 | 2,500,000 | | Ingestion | | | | Direct | | [4,9,31] | | |  |

*Data from human feeding studies; **Data from natural disease outbreaks; - unknown

**Sources:**

1. Centers for Disease Control and Prevention. Alphabetical Index of Parasitic Diseases. Available: http://www.cdc.gov/parasites/az/index.html. Accessed 2 December 2009.

2. Rouquet P, Froment JM, Bermejo M, Kilbourn A, Karesh W, et al. (2005) Wild animal mortality monitoring and human Ebola outbreaks, Gabon and Republic of Congo, 2001-2003. Emerg Infect Dis 11: 283-290.

3. Zampieri CA, Sullivan NJ, Nabel GJ (2007) Immunopathology of highly virulent pathogens: insights from Ebola virus. Nat Immunol 8: 1159-1164. (doi:10.1038/ni1519)

4. Health Canada (2003) Pathogen safety data sheets. Available: http://www.phac-aspc.gc.ca/lab-bio/res/psds-ftss/index-eng.php. Accessed 2 December 2009.

5. Lopman BA, Adak GK, Reacher MH, Brown DWG (2003) Two epidemiologic patterns of Norovirus outbreaks: Surveillance in England and Wales, 1992-2000. Emerg Infect Dis 9: 71-77.

6. Mead PS, Slutsker L, Dietz V, McCaig LF, Bresee JS, et al. (1999) Food-related illness and death in the United States. Emerg Infect Dis 5: 607-625.

7. Katz M, Plotkin SA (1967) Minimal infective dose of attenuated poliovirus for man. Am J Public Health Nations Health 57: 1837-1840.

8. World Health Organization. Publications and Fact sheets. Available: http://www.who.int/research/en/. Accessed 2 December 2009.

9. United States Food and Drug Administration (2003) The Bad Bug Book. Available: http://www.fda.gov/Food/FoodSafety/FoodborneIllness/FoodborneIllnessFoodbornePathogensNaturalToxins/BadBugBook/default.htm. Accessed 2 December 2009.

10. Iftekharul Islam MK, M Shamul Islam Khan (1986) Annotated Bibliography of Asian Literature on Diarrhoeal Diseases. J Diarrhoeal Dis Res 4: 165-208.

11. Fischer TK, Viboud C, Parashar U, Malek M, Steiner C, et al. (2007) Hospitalizations and deaths from diarrhoea and rotavirus among children< 5 years of age in the United States, 1993–2003. J Infect Dis 195: 1117. (doi:10.1086/512863)

12. South Dakota Department of Health. A-Z Topic Index. Available: http://doh.sd.gov/index_a_z.aspx. Accessed 2 December 2009.

13. National Institute of Allergy and Infectious disease. Health and Research Topics A-Z. Available: <http://www.niaid.nih.gov/topics/pages/default.aspx?wt.ac=tnTopics>. Accessed June 2011.

14. Rivera J, Cordero RJB, Nakouzi AS, Frases S, Nicola A, et al. (2010) Bacillus anthracis produces membrane-derived vesicles containing biologically active toxins. P Natl Acad Sci USA 107: 19002-19007. (doi:10.1073/pnas.1008843107)

15. Sewell DL (1995) Laboratory-associated infections and biosafety. Clin Microbiol Rev 8: 389-405.

16. Todar's Online Textbook of Bacteriology (2009). Available: http://www.textbookofbacteriology.net/index.html. Accessed 2 December 2009.

17. Wareing P, Fernandes R (2007) Foodborne bacterial pathogens. Micro-Facts: The Royal Society of Chemistry. pp. 1-167.

18. Wilson M, McNab R, Henderson B (2002) Bacterial Disease Mechanisms: An introduction to cellular microbiology. Cambridge: Cambridge University Press.

19. Fagerlund A, Lindback T, Granum PE (2010) Bacillus cereus cytotoxins Hbl, Nhe and CytK are secreted via the Sec translocation pathway. BMC Microbiol 10: 8. (doi:30410.1186/1471-2180-10-304)

20. The Institute of Food Technologists. Science reports. Available: <http://www.ift.org/knowledge-center/read-ift-publications/science-reports.aspx>. Accessed June 2011.

21. Paddock CD, Sanden GN, Cherry JD, Gal AA, Langston C, et al. (2008) Pathology and pathogenesis of fatal Bordetella pertussis infection in infants. Clin Infect Dis 47: 328-338. (doi:10.1086/589753)

22. Kerr JR, Matthews RC (2000) Bordetella pertussis infection: Pathogenesis, diagnosis, management, and the role of protective immunity. Eur J Clin Microbiol 19: 77-88.

23. Medscape. Infectious Disease Articles. Available: http://emedicine.medscape.com/infectious_diseases. Accessed 2 December 2009.

24. Bossi P TA, Baka A, Van Loock F, Hendriks J, Werner A, Maidhof H, Gouvras G; Task Force on Biological and Chemical Agent Threats, Public Health Directorate, European Commission, Luxembourg. (2004) Bichat guidelines for the clinical management of brucellosis and bioterrorism-related brucellosis. Eurosurveillance 9: E15-E16.

25. Federation of American Scientists. Biological Threat Agents Information. Available: http://www.fas.org/programs/bio/agents.html. Accessed 2 December 2009.

26. Kothary MH, Babu US (2001) Infective dose of foodborne pathogens in volunteers: A review. J Food Safety 21: 49-73.

27. Pollock AM, Whitty PM (1991) Outbreak of Clostridium-perfringens food poisoning. J Hosp Infect 17: 179-186.

28. Scallan E, Hoekstra RM, Angulo FJ, Tauxe RV, Widdowson MA, et al. (2011) Foodborne Illness Acquired in the United States-Major Pathogens. Emerg Infect Dis 17: 7-15. (doi:10.3201/eid1701.P11101)

29. Azad AF (2007) Pathogenic rickettsiae as bioterrorism agents. Clin Infect Dis 45: S52-S55. (doi:10.1086/518147)

30. New Zealand Food Safety Authority (2001) Microbial Pathogens Data Sheets. Available: http://www.foodsafety.govt.nz/elibrary/industry/Staphylococcus_Aureus-Science_Research.pdf. Accessed 2 December 2009

31. Vijgen SMC, Mangen MJM, Kortbeek LM, van Duijnhoven YTHP, AH V (2007) Disease burden and related costs of cryptosporidiosis and giardiasis in the Netherlands. Netherlands: Ministry of Public Health, Welfare and Sports. Available: <http://www.rivm.nl/bibliotheek/rapporten/330081001.pdf>. Accessed 2 December 2009

32. Rasko DA, Rosovitz MJ, Myers GSA, Mongodin EF, Fricke WF, et al. (2008) The pangenome structure of Escherichia coli: Comparative genomic analysis of E-coli commensal and pathogenic isolates. J Bacteriol 190: 6881-6893. (doi:10.1128/jb.00619-08)

33. Molins CR, Carlson JK, Coombs J, Petersen JA (2009) Identification of Francisella tularensis subsp tularensis A1 and A2 infections by real-time polymerase chain reaction. Diagn Micr Infec dis 64: 6-12. (doi:10.1016/j.diagmicrobio.2009.01.006)

34. Carlson PE, Carroll JA, O'Dee DM, Nau GJ (2007) Modulation of virulence factors in Francisella tularensis determines human macrophage responses. Microb Pathogenesis 42: 204-214. (doi:10.1016/j.micpath.2007.02.001)

35. Pechous RD, McCarthy TR, Zahrt TC (2009) Working toward the Future: Insights into Francisella tularensis Pathogenesis and Vaccine Development. Microbiol Mol Biol Rev 73: 684-711. (doi:10.1128/mmbr.00028-09)

36. Berendt RF, Young HW, Allen RG, Knutsen GL (1980) Dose-response of guinea-pigs experimentally infected with aerosols of legionella-pneumophila. J Infect Dis 141: 186-192.

37. Formica N, Yates M, Beers M, Carnie J, Hogg G, et al. (2001) The Impact of Diagnosis by Legionella Urinary Antigen Test on the Epidemiology and Outcomes of Legionnaires' Disease. Epidemiol Infect 127: 275-280.

38. Cascales E, Christie PJ (2003) The versatile bacterial type IV secretion systems. Nat Rev Microbiol 1: 137-149. (doi:10.1038/nrmicro753)

39. Cain KP, Haley CA, Armstrong LR, Garman KN, Wells CD, et al. (2007) Tuberculosis among foreign-born persons in the United States: achieving tuberculosis elimination. Am J Resp Crit Care 175: 75. (doi:10.1164/rccm.200608-1178OC)

40. Western Australia Department of Health (2007) Facts about Pseudomonas aeruginosa in Swimming and Spa Pools Environmental Health Guide. Available: http://www.public.health.wa.gov.au/cproot/1322/2/Facts_about_Pseudomonas_aeruginosa.pdf. Accessed 2 December 2009.

41. Aliaga L, Mediavilla JD, Llosa J, Miranda C, Rosa-Fraile M (2000) Clinical significance of polymicrobial versus monomicrobial bacteremia involving Pseudomonas aeruginosa. Eur J Clin Microbiol 19: 871-874.

42. Brannon MK, Davis JM, Mathias JR, Hall CJ, Emerson JC, et al. (2009) Pseudomonas aeruginosa Type III secretion system interacts with phagocytes to modulate systemic infection of zebrafish embryos. Cell Microbiol 11: 755-768. (doi:10.1111/j.1462-5822.2009.01288.x)

43. Svraka S, Rolain JM, Bechah Y, Gatabazi J, Raoult D (2006) Rickettsia prowazekii and real-time polymerase chain reaction. Emerg Infect Dis 12: 428-432.

44. Edelman R, Levine MM (1986) Summary of an international workshop on typhoid-fever. Rev Infect Dis 8: 329-349.

45. Sayers EW, Barrett T, Benson DA, Bryant SH, Canese K, et al. (2009) Database resources of the National Center for Biotechnology Information (vol 37, pg D5, 2008). Nucleic Acids Res 37: 3124-3124. (doi:10.1093/nar/gkp382)

46. Lake R, Hudson A, Cressey P (2004) Risk Profile: Yersinia enterocolitica in pork. Christchurch: Institute of Environmental Science and Research Limited. Available: <http://www.foodsafety.govt.nz/elibrary/industry/Risk_Profile_Yersinia-Science_Research.pdf>. Accessed 2 December 2009

47. Hacker Jr, Hentschel U, Dobrindt U (2003) Prokaryotic Chromosomes and Disease. Science 301: 790-793. (doi:10.1126/science.1086802)

48. Feigin RD (1992) Textbook of Pediatric Infectious Diseases; Ralph D Feigin JDC, editor: Saunders.

49. Shi L, Albuquerque PC, Lazar-Molnar E, Wang XT, Santambrogio L, et al. (2008) A monoclonal antibody to Histoplasma capsulatum alters the intracellular fate of the fungus in murine macrophages. Eukaryot Cell 7: 1109-1117. (doi:10.1128/ec.00036-08)

50. Yost J (2002) Amebiasis. Pediatr Rev 23: 293-294. (doi:10.1542/pir.23-8-293)

51. Sehgal D, Bhattacharya A, Bhattacharya S (1996) Pathogenesis of infection by Entamoeba histolytica. J Bioscience 21: 423-432

**Table S2: Linear mixed model of minimum infection dose (log transformed)**

| **Fixed Terms** | **Parameter Estimate (β)** | **SE** | **95%LCL** | **95% UCL** | **DF** | **F** | **P** |
| --- | --- | --- | --- | --- | --- | --- | --- |
| Mechanism† Distant  Local | 12.60  3.60 | 1.71  1.06 | 9.11  1.41 | 16.09  5.80 | 1, 40 | 25.79 | **<0.0001** |
| Infection route |  |  |  |  | 2, 38 | 1.58 | 0.22 |
| Transmission mode |  |  |  |  | 1, 34 | 0.08 | 0.39 |
| **Random Terms** | **Variance Component** | **SE** | **95%LCL** | **95% UCL** | **DF** | **LRT** | **P** |
| Kingdom† | 0.00 | 0.00 | 0.00 | 0.00 | 1 | 0.00 | 1.00 |
| Class(kingdom)† | 4.20 | 2.96 | 1.51 | 34.35 | 1 | 3.83 | **0.05** |
| Order (class kingdom)† | 0.00 | 0.00 | 0.00 | 0.00 | 1 | 0.00 | 1.00 |
| Genus (order class kingdom)† | 0.00 | 0.00 | 0.00 | 0.00 | 1 | 0.00 | 1.00 |
| Residual | 17.57 | 4.08 | 11.68 | 29.38 |  |  |  |
| Note: GLMM fitted with a normal error distribution. Parameter estimates are on a log scale and significant values are shown in boldface type. LRT = log-likelihood ratio test. LCL = lower confidence limit, UCL = upper confidence limit. † denotes terms included in final model. N_kingdoms_=4, N_phyla_=11, N_classes_=15, N_orders_=23, N_families_=26, N_genera_=29 N_species_=42. | | | | | | | |

**Table S3: Generalized linear mixed model with Binomial error distribution of case fatality rate**

| **Fixed Terms** | **Parameter Estimate (β)** | **SE** | **95%LCL** | **95% UCL** | **DF** | **F** | **P** | |
| --- | --- | --- | --- | --- | --- | --- | --- | --- |
| Incidence rate (log transformed) |  |  |  |  | 1, 32 | 0.51 | 0.48 | |
| Infection dose (log transformed)† | -0.25 | 0.10 | -0.10 | 0.51 | 1, 38 | 3.94 | **0.05** | |
| Mechanism |  |  |  |  | 1, 31 | 1.32 | 0.26 | |
| Infection route† Ingestion  Inhalation  Wounded skin | 0.01  0.04  0.18 | 0.01  0.03  0.14 | 0.002  0.008  0.03 | 0.05  0.20  0.63 | 2, 26 | 5.30 | **0.01** | |
| Transmission mode |  |  |  |  | 1, 29 | 0.96 | 0.34 | |
| **Random Terms** | **Variance Component** | **SE** | **95%LCL** | **95% UCL** | **DF** | **LRT** | **P** | |
| Kingdom† | 0.00 | 0.00 | 0.00 | 0.00 | 1 | 0.00 | 1.00 | |
| Class(kingdom)† | 1.53 | 1.72 | 0.37 | 144 | 1 | 1.07 | 0.30 | |
| Order (class kingdom)† | 0.00 | 0.00 | 0.00 | 0.00 | 1 | 0.00 | 1.00 | |
| Genus (order class kingdom)† | 1.88 | 1.15 | 0.75 | 10.33 | 1 | 7.97 | **0.005** | |
| Note: GLMM fitted with a normal error distribution. Parameter estimates are back-transformed to the probability scale and significant values are shown in boldface type. LRT = log-likelihood ratio test. LCL = lower confidence limit, UCL = upper confidence limit. † denotes terms included in final model. N_kingdoms_=4, N_phyla_=11, N_classes_=15, N_orders_=23, N_families_=26, N_genera_=29 N_species_=42. | | | | | | | |  |

**Table S4:** Generalized linear mixed model with ordered multinomial error distribution of severity of infection

| **Fixed Terms** | **Parameter Estimate (β)** | **SE** | **95%LCL** | **95% UCL** | **DF** | **F** | **P** | |
| --- | --- | --- | --- | --- | --- | --- | --- | --- |
| Incidence rate (log transformed) |  |  |  |  | 1, 32 | 0.00 | 0.99 | |
| Infection dose (log transformed) |  |  |  |  | 1, 40 | 2.85 | 0.10 | |
| Mechanism |  |  |  |  | 1, 36 | 0.06 | 0.80 | |
| Infection route |  |  |  |  | 2, 37 | 0.75 | 0.48 | |
| Transmission mode |  |  |  |  | 1, 36 | 2.10 | 0.16 | |
| **Random Terms** | **Variance Component** | **SE** | **95%LCL** | **95% UCL** | **DF** | **LRT** | **P** | |
| Kingdom† | 0.51 | 1.00 | 0.07 | 323548 | 1 | 0.40 | 0.53 | |
| Class(kingdom)† | 0.00 | 0.00 | 0.00 | 0.00 | 1 | 0.00 | 1.00 | |
| Order (class kingdom)† | 0.00 | 0.00 | 0.00 | 0.00 | 1 | 0.00 | 1.00 | |
| Genus (order class kingdom)† | 0.62 | 0.84 | 0.13 | 368 | 1 | 0.88 | 0.35 | |
| Note: GLMM fitted with a normal error distribution. Parameter estimates are back-transformed to the probability scale and significant values are shown in boldface type. LRT = log-likelihood ratio test. LCL = lower confidence limit, UCL = upper confidence limit. † denotes terms included in final model. N_kingdoms_=4, N_phyla_=11, N_classes_=15, N_orders_=23, N_families_=26, N_genera_=29 N_species_=42. | | | | | | | |  |
